# Biomechanics in Sport and Motion Analysis

**DOI:** 10.3390/bioengineering13050575

**Published:** 2026-05-19

**Authors:** Rajat Emanuel Singh, Kamran Iqbal

**Affiliations:** 1 Department of Kinesiology and Engineering, Northwestern College, Orange City, IA 51041, USA; rajat.singh@nwciowa.edu; 2 School of Engineering and Engineering Technology, University of Arkansas at Little Rock, Little Rock, AR 72204, USA

## 1. Introduction

In sports science, there is a growing demand to apply the principles of classical and modern physics to understand human movement, prevent injuries, and enhance performance [1,2]. Technological advancements over the past century have created opportunities for clinicians, practitioners, researchers, and engineers to address problems related to human movement, particularly in sports science and sports medicine [2]. However, significant gaps remain across multiple subfields of sports science and motion analysis. Motivated by this need, we called for original research articles, short communications, review articles, and opinion pieces for this Special Issue to address some of the most pressing questions in these and related fields. We have compiled six articles that tackle these gaps in the current literature, supported by novel research, with a particular focus on the lower extremity. What distinguishes this collection is not only the thematic coherence around lower-extremity biomechanics but also the populations studied, from recreational runners and novice soccer players to patients with diabetic peripheral neuropathy (DPN), and the diverse methodological approaches employed, spanning wearable accelerometers, markerless motion capture, machine learning algorithms, and vector coding analysis.

## 2. Special Issue Articles

There are a few studies examining acceleration spike asymmetries resulting from running-induced fatigue. Such fatigue typically affects several kinematic parameters and may increase injury risk. Delgado-García et al. investigated the relationship between lower-limb acceleration spike asymmetries and fatigue using inertial measurement units and photogrammetry [3]. In this study, 18 recreational runners were recruited and asked to run on a treadmill for 30 min to induce fatigue. Accelerometer data were collected from the tibia and sacrum to quantify acceleration spikes for both legs. The study found that fatigue affected the dominant and non-dominant legs differently, with an increase in acceleration spikes in the dominant leg compared to the non-dominant leg. The authors further suggested that this increase may reflect greater mechanical loading and potential overuse, thereby increasing injury risk, and emphasized that kinematic data should be acquired from both limbs in research studies.

Feng et al. developed two neural network models, a convolutional neural network (CNN) and a multilayer perceptron (MLP), to estimate three-dimensional ground reaction forces (GRFs) and center of pressure (CoP) trajectories along the mediolateral (x), vertical (y), and anteroposterior (z) directions using kinematic data from markerless motion capture during gait [4]. Model performance was evaluated using the correlation coefficient (r) and the relative root mean square error (rRMSE), yielding high precision. Moreover, the CNN appeared to outperform the MLP in estimating the mediolateral components of GRF and CoP during walking.

The issues in movement mechanics are not limited to continuous tasks such as walking and running. There are also several gaps in the literature regarding discrete sports-related skills. A study by Gallego-Pérez et al. addresses one such gap by examining a discrete task, the countermovement jump (CMJ) [5]. They recruited thirty-one participants and compared muscle activation of the vastus medialis (VM), vastus lateralis (VL), and biceps femoris (BF) during a single CMJ and five consecutive CMJs. They found no evidence of increased fatigue in five consecutive CMJs compared to a single CMJ. The VM and VL exhibited higher activation in the take-off phase than in the landing phase, whereas the BF showed similar activation across phases. However, the BF demonstrated a notable difference in the landing phase under the five consecutive CMJ conditions.

Ullah et al. investigated the effects of DPN on balance by evaluating gait kinematic and kinetic parameters during overground walking [6]. They assessed static stability, dynamic stability, and mobility using the tandem test, center-of-pressure Time-to-Contact (TTC) with the mediolateral (ML) stability boundary, and the Timed Up and Go (TUG) test, respectively. Additionally, other gait parameters, such as velocity and number of steps, were analyzed. The study found no significant differences in dynamic stability; however, patients with DPN exhibited slower gait, shorter steps, poorer tandem test performance, and slower TUG performance. Overall, these findings suggest that patients with DPN may compensate by modifying their gait parameters to preserve dynamic stability control.

Proficiency is a state that requires further investigation, particularly in terms of segmental coordination within sports biomechanics. Zhang et al. conducted a comparative study between experienced athletes and novices during soccer instep kicking, recruiting 14 collegiate-level athletes and 32 novices [7]. The authors quantified inter-segmental coordination using hip–knee and knee–ankle coupling patterns via vector coding and computed agonist–antagonist muscle activation ratios of leg muscles. The study revealed distinct coordination and neuromuscular patterns between experienced and novice participants. Experienced athletes demonstrated a greater percentage of knee–ankle (shank-dominant) coordination. Additionally, athletes achieving higher ball speeds exhibited more knee flexion–dominant patterns during the backswing and leg-cocking phases, and more knee extension–dominant patterns during the acceleration phase. Furthermore, lower activation ratios of the tibialis anterior and gastrocnemius were associated with improved kicking accuracy. These findings suggest that refined distal segment coordination and optimized muscle activation strategies contribute to enhanced kicking performance.

Furthermore, Zhang et al., in a separate study, investigated different training types (strength plus kick training, coordination plus kick training, and kick-only training) over an eight-week period, conducted three times per week, to determine which method may improve inter-segmental coordination and accuracy during soccer instep kicking [8]. Thirty-two participants were randomly assigned to these groups. The increase in ball velocity was not specific to any one training type, as all three groups demonstrated significant improvements. However, the coordination training group showed more changes in kinematic and neuromuscular patterns among novices. For instance, the knee–ankle (shank phase) coordination pattern time increased during the leg cocking phase, while the hip–knee (thigh-phase) coordination pattern time reduced significantly during the back swing. Moreover, a significant reduction in the relative activity of the tibialis anterior and gastrocnemius muscles was observed exclusively in the coordination training group, suggesting improvements in kicking accuracy and neuromuscular control.

A comprehensive summary of these six articles is provided in Table 1.

## 3. Conclusions

Our Special Issue successfully covers six articles emphasizing the lower extremity. These are original research articles that provide both rehabilitation and training approaches to improve neuromuscular and neuromechanical control. Moreover, this Special Issue also presents technical methods for estimating kinetics from kinematic features. This series of articles should be beneficial for training athletic populations and for rehabilitation in clinical populations, using a variety of methods ranging from sports medicine to clinical rehabilitation protocols. The current issue focuses exclusively on lower-extremity-related research; in future Special Issues, upper-extremity methods should also be emphasized.

## Figures and Tables

**Table 1:** Summary of articles in Special Issue (Biomechanics in Sport and Motion Analysis, 2025–2026).

Study (Author, Year)	Objective	Participants	Methodology	Key Findings
Delgado-García et al. [3]	Evaluate effects of a 30 min run on lower limb acceleration spikes’ asymmetries.	18 recreational runners (35.6 ± 7.5 years).	Treadmill protocol using accelerometers (tibias/sacrum) and photogrammetry.	Right tibial acceleration spikes increased; tibial load asymmetry rose from 9% to 25%.
Feng et al. [4]	Develop neural network models to estimate 3D GRF and COP during walking.	146 college students: 62 males (age: 20.3 ± 1.2 years) and 94 females (age: 19.8 ± 1.4 years).	Markerless motion capture with MLP and CNN models.	High correlation (r > 0.9) for GRF; CNN outperformed MLP in estimating COP.
Gallego-Pérez et al. [5]	Study normative muscle activation (VL, VM, BF) during single and five consecutive CMJs.	31 participants (20 F, 11 M; avg. 22.5 ± 3.3 years).	Cross-sectional descriptive study using surface electromyography (EMG).	VM/VL activation was higher during take-off compared to landing; five jumps did not induce greater fatigue than one jump.
Ullah et al. [6]	Investigate gait/postural deficits in DPN patients.	15 DPN patients and 15 healthy controls.	Overground walking with motion capture, force platforms, and TUG tests.	DPN patients had slower gait and shorter steps; significant static balance deficits were observed.
Zhang et al. [7]	Compare inter-segmental coordination during soccer instep kicking (experienced vs. novices).	14 experienced athletes and 32 novices.	Motion capture and EMG analysis using non-linear vector coding.	Athletes showed greater knee–ankle shank dominance; lower muscle ratios linked to higher accuracy.
Zhang et al. [8]	Determine effects of 8-week coordination vs. strength training on novice instep kicking.	32 male college novices (randomly assigned). An 8-week training intervention (3×/week) assessed via vector coding and EMG.		All groups increased ball speed; coordination training specifically improved accuracy and control.
